# Mechanical Efficiency at Different Exercise Intensities Among Adolescent Boys With Different Body Fat Levels

**DOI:** 10.3389/fphys.2019.00265

**Published:** 2019-03-15

**Authors:** Georges Jabbour, Lina Majed

**Affiliations:** Sport Science Program, College of Arts and Sciences, Qatar University, Doha, Qatar

**Keywords:** mechanical efficiency, energy consumption, obese, body mass, catecholamine, incremental cycle test to exhaustion, adolescent boys

## Abstract

This study investigated the mechanical efficiency (ME) and associated factors in obese, overweight, and normal-weight adolescent boys during incremental cycle exercise test to exhaustion. Forty-five sedentary adolescent boys (13–14 years old) were separated in three groups according to the percentage of fat mass as follows: 15 normal-weight (NW) (body fat: 16.0 ± 1.9%), 15 overweight (OW) (body fat: 24.0 ± 1.6%), and 15 obese (OB) (body fat: 31.0 ± 3.0%). All groups completed an incremental cycle exercise to exhaustion in which energy consumption (E, W), ME (%), lipid oxidation rate (LO, %), plasma epinephrine and norepinephrine concentrations were determined consecutively at rest and at three intensity levels corresponding to 50 and 75% of each participant’s maximal heart rate (50%HRmax and 75%HRmax) and peak oxygen consumption ($\overset{˙}{V}$O_2peak_). During the incremental cycle exercise test, plasma epinephrine, and norepinephrine responses as well as ME determined at 50%HRmax, 75%HRmax, and at VO_2peak_ stages were significantly lower in OB compared to NW and OW individuals (*ps* < 0.01). Multiple linear regressions showed that body weight (ß = -0.64, *p* < 0.001), energy consumption (ß = -0.24, *p* < 0.05) and lipid oxidation (ß = 0.69, *p* < 0.01) were significant predictors of ME at 50%HRmax. However, at 75%HRmax and $\overset{˙}{V}$O_2peak_, significant predictors of ME were epinephrine (ß = 0.34, ß = 0.49, respectively, *ps* = 0.01), norepinephrine (ß = 0.26, ß = 0.60, respectively, *ps* < 0.05) and power output (ß = 0.62, ß = 0.71, respectively, *ps* < 0.01). These findings suggest that excess in body weight exerts a negative effect on ME at a low intensity by increasing energy consumption for obese and overweight adolescent boys, while at higher intensities (75%HRmax and VO_2peak_) the lower ME could be better explained by the lower power output and catecholamine responses that were attenuated among obese and overweight adolescent boys.

## Introduction

Mechanical efficiency (ME) refers to the ability of an individual to transfer the energy consumed into performing external work (Weinstein et al., 2004). ME has recently been investigated as a potential factor underlying metabolic and mechanical adaptations to exercise not only among trained subjects (Boone et al., 2010) but also in special populations (Jabbour et al., 2013, 2017; Jabbour and Iancu, 2015). In parallel to other “classical” variables such as cardiovascular risk factors, quality of life, maximal oxygen consumption, ME has been examined as a source of information regarding the effectiveness of exercise interventions (Villelabeitia-Jaureguizar et al., 2018). With this growing interest in using ME for performance and health evaluations there is still a lot to know about underlying key factors.

ME has been proposed as an important measure relating to weight loss and obesity. Indeed, it was suggested that ME is influenced by body weight status (Butte et al., 2007) and metabolic milieu (Jabbour and Iancu, 2015; Laaksonen et al., 2018). A decreased ME may be considered as a limitation for physical activity (Layec et al., 2011; Jabbour and Iancu, 2015), where less efficiency for a given work output is attributed to higher energy consumption and energy cost of breathing during exercise (Layec et al., 2011; Jabbour and Iancu, 2015). For Lafortuna et al. (2006), the decreased ME reported in obese adults may be related to the increased proportion of glycolytic muscle fibers (Kriketos et al., 1997) which are substantially less efficient compared to type I fibers. The latter interpretation was proposed to explain the higher cycling energy cost (Coyle et al., 1992) found in obese adults as compared to normal weight and overweight adults. For Butte et al. (2007), the lower ME values observed for overweight children may be the consequence of the excess in body mass that may limit muscle efficiency. However, the study of Jabbour et al. (2013) conducted on 660 children showed that ME was not affected by body weight status. For these authors, the contradictory lower ME observed in previous studies may be simply related to the method used for ME calculation (net vs. crude value).

More recently, Laaksonen et al. (2018) have investigated the relationship between muscle metabolism and ME among 17 healthy recreationally active male subjects at an intensity corresponding to 45% of VO_2peak_. Their findings suggested that the use of plasma fatty acids was higher in more efficient subjects and correlated significantly with ME. Non-etheless, no significant differences for blood glucose concentration were observed between the groups suggesting that plasma fatty acids may be an important determinant of ME during submaximal exercises. Furthermore, an interventional study of Jabbour and Iancu (2015) reported an increase in ME following a high intensity training program which was linked to improved homeostasis model assessment estimated insulin resistance and concomitant increases in power output. Interestingly, these improvements were reported at higher intensity stages of an incremental maximal cycling test corresponding to 60%, 80%, and 100% of peak power, respectively. As a factor of performance, ME may be involved in both aerobic and anaerobic performance (Kriketos et al., 1997; Jabbour and Iancu, 2015; Jabbour et al., 2017), therefore indicating that key factors underlying ME may diverge depending on the intensity and task performed.

Most studies on factors contributing to ME in obese individuals have looked into skeletal muscle adaptations and how they relate to metabolic improvements with training that have been associated for instance with muscle strengthening (Jabbour and Iancu, 2015). However, changes in ME may also be related to hormonal adaptations such as epinephrine and norepinephrine responses. Human studies indicate that obese individuals have reduced catecholamine responses (Jabbour et al., 2011; Vettor et al., 1997) which could significantly affect exercise performance (Strobel et al., 1999). Therefore, the first aim of the present study was to compare ME, metabolic and physiological responses between normal weight, overweight and obese adolescent boys at different intensity levels of an incremental cycle exercise test to exhaustion. The second aim was to examine the relationship between ME and potential underlying factors amongst which plasma epinephrine and norepinephrine responses. We expect to find the common ME, metabolic and physiological differences as previously reported between the normal weight and obese groups during exercise. As for the relationship between ME and underlying factors, we hypothesize that it would be intensity-dependent and that efficiency would be positively correlated to epinephrine and norepinephrine responses during exercise.

## Materials and Methods

### Participants

Forty-five healthy adolescent boys were recruited from several high schools in Lebanon. To prevent any maturation variability, only participants in the age range of 13–14 years who were at the same Tanner stage (Stage 3) (Tanner, 1962) were selected. Further inclusion criteria for participation included (i) being sedentary [participating in <1 h per week of structured exercise, as assessed by the International Physical Activity Questionnaire (Craig et al., 2003)], (ii) presenting no metabolic, cardiovascular or chronic health problems, (iii) having no history of drug consumption, or (iv) smoking. Health-related information was obtained from the participants’ family physician prior to the study. Volunteers were separated into three groups based on the percent body fat (%body fat) criterion previously described by McCarthy et al. (2006): a normal-weight (NW) group (*n* = 15; %body fat <22%), an overweight (OW) group (*n* = 15; %body fat = 22–25%), and an obese (OB) group (*n* = 15; %body fat >26%). Participants’ physical characteristics and aerobic fitness level are presented in Table 1. Before the start of the experiment, a written informed consent was obtained from the parents and adolescents were familiarized with all testing equipment and procedures. The whole study was approved by the Ethical Committee on Human Research (ECHR) of the University of Balamand (Lebanon) according to the declaration of Helsinki.

**Table 1:** Physical characteristics and aerobic fitness of participants in the three groups: normal weight (NW), overweight (OW), and obese (OB) adolescents.

	NW (*N* = 15)	OW (*N* = 15)	OB (*N* = 15)	Group effect *(df = 2)*	
				
				*F*	*p*
Age (years)	13.6 (0.1)	13.4 (0.1)	13.6 (0.3)	1.7	0.31
Height (cm)	162.9 (6.2)	164.4 (10.4)	168.9 (9.6)	1.6	0.33
Body mass (kg)	50.5 (5.2)	67.0 (10.0)^a^	88.7 (14.7)^a,b^	11.2	<0.0001
BMI (kg⋅m^-2^)	18.9 (1.1)	24.5 (1.5)^a^	30.8 (2.3)^a,b^	19.1	<0.0001
FM (%)	16.0 (1.9)	24.5 (1.6)^a^	31.0 (3.0)^a,b^	23.9	<0.0001
FFM (kg)	43.0 (6.0)	52.0 (9.0)^a^	62.0 (8.0)^a,b^	21.4	<0.0001
$\overset{˙}{V}$O_2_peak (L⋅min^-1^)	2.10 (0.12)	2.36 (0.09)^a^	2.43 (0.11)^a^	11.8	<0.0001


*Values are presented as mean (standard deviation). BMI, body mass index; FM, fat mass; FFM, fat free mass; $\overset{˙}{V}$O_*2*_peak, peak value of oxygen consumption. ^*a*^Significant difference with NW (*p* < 0.01). ^*b*^Significant difference with OW (*p* < 0.01).*

### Protocol and Materials

After an overnight fast, participants reported once to the laboratory to perform the protocol that lasted 1 h on average. They were asked to refrain from strenuous exercise 24 h before the test. Anthropometric characteristics were firstly measured to assign participants to a weight category group, after which an incremental cycle exercise test followed.

### Anthropometric Measurements

Body mass was measured to the nearest 0.1 kg, with the participants wearing light clothing without shoes, using an electronic scale (MFB 150K100, Kern, Germany). Height was determined to the nearest 0.5 cm with a measuring tape fixed to the wall. The body mass index (BMI, kg⋅m^-2^) was calculated as the ratio of body mass (kg) to height squared (m^2^). The %body fat, referred to here as fat mass (FM, %) was estimated from 3 skinfold thickness measurement sites (biceps, triceps, and sub-scapular) according to the validated method of Slaughter et al. (1988) for children and youth. The fat free mass (FFM, kg) was calculated by subtracting the fat mass from the body mass.

### Incremental Cycle Exercise Test to Exhaustion

Participants performed a maximal test on an upright cycle ergometer (Monark Ergomedic 839E, Monark, Sweden) to determine their peak oxygen consumption ($\overset{˙}{V}$O_2_peak). A breath-to-breath automated metabolic system (CPX, Medical Graphics, St-Paul, MN, United States) was used to collect gas exchange data. Prior to each test, the system was calibrated according to the manufacturer’s instructions using standard gasses of known concentration as well as a calibration syringe for air flow. The laboratory environment was controlled where temperature and relative air humidity were maintained around 23 C and 60%, respectively. Heart rate was continuously measured using a heart rate monitor (Polar-F6, Polar, Finland). At the start of the test, participants remained seated for 5 min on the bicycle ergometer to measure their resting values. The test started at an initial power of 60 W and progressively increased by 20 W every 2 min until exhaustion. During the test, adolescents were instructed to pedal at a rate of 50–70 revolutions per minute that was monitored using an electronic counter (MEV 2000) embedded in the ergocycle. The test was terminated when adolescents could no longer maintain the required pedaling rate (<40 revolutions per minute) or requested to stop the exercise. At the end of the protocol, participants were asked to perform an active recovery of 5 min at 25 W.

At rest and at the end of each intensity level, a venous blood sample was collected from the antecubital vein in a vacutainer tube containing Ethylene Diamine Tetra Acetic Acid (EDTA). Plasma from the venous blood samples was separated by centrifugation at 3000 × *g* for 20 min (4°C) (ORTO ALRESA mod. Digicen.R, Spain). Aliquots were immediately frozen and stored at -80°C for use in subsequent chemical analyses. At the end of incremental test and after a 3-min recovery period, fingertip capillary blood samples were collected and immediately analyzed for blood lactate concentration using a Lactate Pro portable device (Arkray, Japan). This procedure was done to verify one of the test termination criteria.

### Data Analysis

#### Calculation of Metabolic and Physiological Variables

Gas exchange data were collected on a breath-to-breath basis with a continuous and synchronized measurement of heart rate (HR, beats⋅min^-1^). Mean values of HR, oxygen consumption ($\overset{˙}{V}$O_2_, L⋅min^-1^), carbon dioxide production ($\overset{˙}{V}$CO_2_, L⋅min^-1^), and respiratory exchange ratio (RER) were computed as the average of the last 20 s of each intensity level where a steady-state was reached. $\overset{˙}{V}$O_2_peak was achieved when participants fulfilled at least three of the following criteria: a peak or plateau in $\overset{˙}{V}$O_2_ values despite an increase in exercise intensity, a RER greater than 1.1, a peak HR above 90% of the predicted maximal HR (220-age), a blood lactate concentration higher than 8.0 mmol⋅L^-1^ and the apparent exhaustion of the subject (Spiro, 1979). In the present work, main variables were assessed at rest and at three stages corresponding to 50 and 75% of each participant’s maximal heart rate (50%HRmax and 75%HRmax) and at the peak oxygen consumption ($\overset{˙}{V}$O_2_peak) level.

Substrate oxidation was determined at the submaximal aerobic intensity stages (50%HRmax and 75%HRmax) based on the corresponding mean values of the non-protein RER. Specifically, the percentage of lipid oxidation (%LO) contributing to energy was calculated using the method of McGilvery and Goldstein (1983) as follows: %LO = [(1 – RER)/0.29] × 100. The percentage of carbohydrate oxidation (%CHO) was then deduced by subtracting the %LO from 100.

#### Calculation of Mechanical Efficiency

Net mechanical efficiency (ME_net_, %) was calculated using the formula developed by Lafortuna et al. (2006) as the ratio of work performed (W) to the rate of energy consumed (E, W) above resting level, that was in turn computed as follows: *E* = (4.94 RER + 16.04) × $\overset{˙}{V}$O_2net_ / 60 (Garby and Astrup, 1987). Net $\overset{˙}{V}$O_2_ ($\overset{˙}{V}$O_2net_, L⋅min^-1^) was calculated by subtracting the resting value from the gross value at each intensity stage. The resting values of E (E_rest_) were also determined based on the equation using $\overset{˙}{V}$O_2rest_ values instead of the $\overset{˙}{V}$O_2net_ values.

#### Blood Analyses

Plasma epinephrine and norepinephrine concentrations were measured using high-performance liquid chromatography (HPLC) (Chromsystems, Thermo finnigan, France), following the method of Koubi et al. (1991). Before the HPLC run, catecholamines were extracted by selective absorption from sodium bisulfite (Chromsystems-HPLC-Kit, Waters, Milford, MA, United States). 1 mL of plasma previously centrifuged was shaken up briefly with 250 μL of sodium bisulfite (0.25%) and 50 μL internal standard solution (600 pg dihydroxybenzylamine). After a three times wash (solution TRIS 1M EDTA, pH 8.8), catecholamines were eluted with 120 μL buffer (10 mL ultra-pure water, 130 μL acetic acid, 100 μL bisulfate 0.25% and 25 μL EDTA 10%). Eluant was centrifuged at 4000 rpm for 10 min (Thermo Fisher Scientific, Jouan. GR412), after which a 50 μL of the sample eluant was injected into HPLC column (Column Waters reference 5007 alumina 20 mg) and eluted with a mobile phase. The flow rate was 1 mL⋅min^-1^ at 13.8 mPa and a potential of 0.60 V. The chromatogram was analyzed by computer integration (Baseline 815, Waters). The detection limit of catecholamines in the described method was 0.06 nM and the inter-assay coefficient of variation was 6.5%. The blood lactate concentration was determined enzymatically using a lactate analyzer (Microzyme, Cetrix, France). Plasma hormones and lactate values were corrected for plasma volume changes using the equation of Van Beaumont (1972).

### Statistical Analysis

Data are presented as mean and standard deviation (SD). After testing for normal distribution (Kolmogorov–Smirnov test), differences between the three groups (i.e., NW, OW, and OB) were analyzed using a one-way analysis of variance (ANOVA) performed on all dependent variables at rest, 50%HRmax, 75%HRmax, and $\overset{˙}{V}$O_2peak_. A two-way ANOVA was performed to further test the interaction effect between the groups and the three relative exercise intensity levels on ME values. Each repeated measures ANOVA was preceded by a Mauchly’s sphericity test, and if the test was significant (indicating a violation of the hypothesis of variance homogeneity), a Huynh-Feldt correction procedure was used to adjust the degrees of freedom. When needed, a *post hoc* analysis using Newman-Keul’s test was performed for pairwise comparisons. A multiple regression analysis was conducted at each of the studied intensity level (i.e., 50%HRmax, 75%HRmax, and $\overset{˙}{V}$O_2peak_) to examine the relationship between the ME_net_ and various potential predictors (i.e., body weight, power output, energy consumption, lipid oxidation, epinephrine, norepinephrine). The analyses were performed using IBM SPSS Statistics 19 software (IBM SPSS Statistics for Windows, Version 24.0, Armonk, NY, United States: IBM Corp.). A value of *p* < 0.05 was accepted as the minimal level of statistical significance.

## Results

### Physical Characteristics and Aerobic Fitness Levels

The age and body height did not differ significantly between the groups of adolescents (Table 1). As expected, results indicated significantly different body mass, BMI and %FM between each of the three groups (*ps* < 0.01). Furthermore, the FFM (kg) was significantly higher for OB in comparison to OW and NW as well as for OW in comparison to NW (*ps* < 0.01). The maximal aerobic capacity (absolute $\overset{˙}{V}$O_2_peak, L⋅min^-1^) was significantly higher for the OB and OW groups as compared to the NW group (*ps* < 0.01).

### Mechanical Efficiency, Metabolic and Physiological Variables

#### Resting Values

Resting values of oxygen consumption were significantly higher for the OB group in comparison to the NW and OW groups, as well as for the OW as compared to the NW (*p* < 0.01, Table 2). However, no significant differences were revealed between groups for resting values of lactate concentration, heart rate, RER, and epinephrine and norepinephrine concentrations.

**Table 2:** Metabolic and physiological responses at rest for the three groups: normal weight (NW), overweight (OW), and obese (OB) adolescents.

	NW (*N* = 15)	OW (*N* = 15)	OB (*N* = 15)	Group effect *(df* = 2)	
				
				*F*	*p*
$\overset{˙}{V}$O_2rest_ (L⋅min^-1^)	0.19 (0.01)	0.35 (0.02)^a^	0.45 (0.02)^a,b^	11.8	<0.0001
Lactate (mmol⋅L^-1^)	1.3 (0.3)	1.3 (0.5)	1.3 (0.7)	1.3	1.53
HR (beats⋅min^-1^)	78.3 (2.2)	76.2 (9.1)	77.4 (3.1)	2.9	0.44
RER	0.78 (0.09)	0.79 (0.09)	0.78 (0.09)	2.2	0.22
E_rest_ (W)	69 (11)	79 (17)^a^	99 (15)^a,b^	6.6	<0.01
Epinephrines (nmol⋅L^-1^)	0.85 (0.01)	0.81 (0.02)	0.82 (0.01)	1.9	0.24
Norepinephrines (nmol⋅L^-1^)	2.50 (0.01)	2.40 (0.04)	2.60 (0.03)	3.9	0.74


*Values are presented as mean (standard deviation). $\overset{˙}{V}$O_*2rest*_: oxygen consumption at rest, HR: heart rate, RER: respiratory exchange ratio, E: energy consumption in Watts. ^*a*^Significant difference with NW (*p* < 0.01). ^*b*^Significant difference with OW (*p* < 0.01).*

#### Submaximal Exercise Intensities

At the studied moderate intensity (i.e., 50%HRmax), most of the variables differed significantly between each of the three groups even though this relative intensity induced similar values of power output (PO), heart rate and lactate concentrations in all groups (Table 3). For instance, higher oxygen and energy consumption, RER and %LO and lower %CHO, epinephrine and norepinephrine concentrations were seen in the OB group as compared to the OW and NW groups (*ps* < 0.01), as well as for the OW in comparison to the NW group (*ps* < 0.01).

**Table 3:** Metabolic and physiological responses at the studied submaximal intensity levels (50%HRmax, 75%HRmax) of the incremental cycle exercise test to exhaustion for the three groups: normal weight (NW), overweight (OW), and obese (OB) adolescents.

	NW (*N* = 15)	OW (*N* = 15)	OB (*N* = 15)	Group effect *(df* = 2)	
				
				*F*	*p*
**At 50%HRmax**					
$\overset{˙}{V}$O_2net_ (L⋅min^-1^)	1.32 (0.02)	1.68 (0.02)^a^	1.81 (0.02)^a,b^	42.1	<0.0001
PO (W)	80 (5)	80 (10)	80 (10)	1.3	0.31
Lactate (mmol⋅L^-1^)	3.9 (1.9)	3.8 (1.7)	4.1 (1.1)	2.6	0.36
HR (beats⋅min^-1^)	104.7 (2.1)	106.2 (3.1)	102.9 (6.1)	0.6	0.54
RER	0.78 (0.05)	0.82 (0.04)^a^	0.89 (0.07)^a,b^	11.2	<0.01
%LO	48 (2)	39 (1)^a^	22 (3)^a,b^	20.2	<0.0001
%CHO	52 (2)	61 (1)^a^	78 (3)^a,b^	20.1	<0.0001
E(W)	458 (11)	562 (32)^a^	615 (14)^a,b^	30.1	<0.0001
Epinephrines (nmol⋅L^-1^)	1.61 (0.1)	0.98 (0.2)^a^	0.82 (0.1)^a,b^	8.6	<0.01
Norepinephrines (nmol⋅L^-^)	5.4 (0.3)	4.2 (0.3)^a^	3.9 (0.4)^a,b^	19.4	<0.001
**At 75%HRmax**					
$\overset{˙}{V}$O_2net_ (L⋅min^-1^)	2.01 (0.04)	2.02 (0.02)	2.01 (0.03)	2.1	0.22
PO (W)	140 (5)	120 (10)^a^	120 (10)^a^	11.8	<0.001
Lactate (mmol⋅L^-1^)	6.9 (1.9)	6.8 (1.7)	6.1 (1.1)	2.6	0.36
HR (beats⋅min^-1^)	144.2 (2.2)	152.2 (2.1)	148.9 (2.1)	0.9	0.56
RER	0.89 (0.05)	0.87 (0.04)	0.87 (0.07)	1.6	0.64
%LO	8 (2)	9 (1)	8 (3)	11.2	0.46
%CHO	92 (2)	91 (1)	92 (3)	3.1	1.1
E (W)	685 (12)	684 (12)	681 (19)	2.1	2.2
Epinephrines (nmol⋅L^-1^)	2.01 (0.1)	1.81 (0.2)	1.51 (0.4)^a,b^	6.6	<0.01
Norepinephrines (nmol⋅L^-1^)	7.4 (0.2)	5.9 (0.3)^a^	5.3 (0.1)^a,b^	11.1	<0.001


*Values are presented as mean (standard deviation). $\overset{˙}{V}$O_*2net*_: oxygen consumption above the resting level, PO: power output, HR: heart rate, RER: respiratory exchange ratio, %LO: percent contribution of lipid oxidation to energy, %CHO: percent contribution of carbohydrate oxidation to energy, E: energy consumption. ^*a*^Significant difference with NW (*p* < 0.01). ^*b*^Significant difference with OW (*p* < 0.01).*

At 75%HRmax, the oxygen and energy consumption values as well as the heart rate, RER and lactate concentrations did not differ statistically between the three groups although power output was significantly higher for the NW group as compared to both OW and OB groups (Table 3). Moreover, the OB group showed a significantly lower increase in epinephrine and norepinephrine compared to the NW and OW groups (*ps* < 0.01).

#### Peak Exercise Intensity Values

At $\overset{˙}{V}$O_2peak_, all studied variables differed significantly between each of the three groups, except for heart rate and RER values (Table 4). Specifically, the NW group reached the highest power output $\overset{˙}{V}$ and presented the lowest $\overset{˙}{V}$O_2peak_ and lactate concentration as compared to the OW and OB groups (*ps* < 0.01). The OB and OW groups presented significantly different epinephrine and norepinephrine levels that were lower when compared to the NW groups (*ps* < 0.01).

**Table 4:** Metabolic and physiological responses at the peak intensity level ($\overset{˙}{V}$O_2_peak) of the incremental cycle exercise test to exhaustion for the three groups: normal weight (NW), overweight (OW), and obese (OB) adolescents.

	NW (*N* = 15)	OW (*N* = 15)	OB (*N* = 15)	Group effect (*df* = 2)	
				
				*F*	*p*
$\overset{˙}{V}$O_2peak_ (L⋅min^-1^)	2.10 (0.12)	2.36 (0.09)^a^	2.43 (0.11)^a^	11.8	<0.0001
PO (W)	180 (19)	160 (10)^a^	160 (15)^a^	19.8	<0.001
Lactate (mmol⋅L^-1^)	8.4 (4.4)	8.9 (3.4)	9.8 (3.3)^a,b^	21.32	<0.001
HR (beats⋅min^-1^)	202.9 (4.1)	201.2 (3.7)	201.3 (4.1)	0.4	0.64
RER	1.22 (0.05)	1.18(0.03)	1.20 (0.04)	1.21	0.29
E (W)	780 (38)	860 (22)^a^	880 (19)^a^	4.1	<0.01
Epinephrines (nmol⋅L^-1^)	2.78 (0.3)	2.01 (0.3)^a^	1.78 (0.3)^a,b^	8.6	<0.01
Norepinephrines(nmol⋅L^-1^)	13.4 (0.2)	12.1 (0.2)^a^	11.3 (0.2)^a,b^	19.4	<0.001


*Values are presented as mean (standard deviation). $\overset{˙}{V}$O_*2*_peak: peak oxygen consumption above the resting level, PO: power output, HR: heart rate, RER: respiratory exchange ratio, E: energy consumption. ^*a*^Significant difference with NW (*p* < 0.01). ^*b*^Significant difference with OW (*p* < 0.01).*

#### Mechanical Efficiency

**FIGURE 1:**
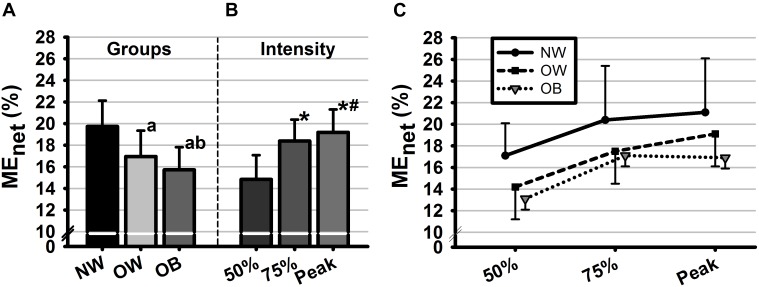
Mean values of net mechanical efficiency (ME_net_, %) as a function of **(A)** groups, **(B)** intensities, and **(C)** their interaction. Groups are defined in relation to weight status as normal weight (NW), overweight (OW) and obese (OB) and intensities are defined in reference to HR_max_ as 50%HR_max_, 50%HR_max_, and 100%HR_max_. Error bars represent the standard deviation. ^a^Significant difference with NW (*p* < 0.01). ^b^Significant difference with OW (*p* < 0.01). ^∗^Significant difference with 50%HR_max_. ^#^Significant difference with 75%HR_max_.

The two-way ANOVA on ME_net_ revealed significant main effects of group [*F*(2, 28) = 102.95, *p* < 0.001, *η*^2^ = 0.88] and intensity [*F*(2, 28) = 137.03, *p* < 0.001, *η*^2^ = 0.91]. No significant interaction effect was found between factors. *Post hoc* comparisons showed that ME_net_ differed significant between all groups and all intensities (*ps* < 0.01) with the smallest values found for the OB group as well as for the lowest studied intensity level (Figure 1).

Multiple linear regressions were calculated to examine the degree to which studied variables predicted ME_net_ at each studied intensity level. Significant regression equations were found at 50%HRmax [*F*(2,42) = 20.25, *p* < 0.01, *R*^2^ = 0.47], 75%HRmax [*F*(2,42) = 4.14, *p* < 0.05, *R*^2^ = 0.19] and $\overset{˙}{V}$O_2peak_ [*F*(3,35) = 11.01, *p* < 0.01, *R*^2^ = 0.48]. Specifically, at 50%HRmax, body weight (ß = -0.64, *p* < 0.001), energy consumption (ß = -0.24, *p* < 0.05) and lipid oxidation (ß = 0.69, *p* < 0.01) were significant predictors of ME_net_. At 75%HRmax, the analysis demonstrated that the epinephrine (ß = 0.34, *p* = 0.01), norepinephrine (ß = 0.26, *p* = 0.01) and power output (ß = 0.62, *p* < 0.01) contributed significantly to ME_net_. Finally, at $\overset{˙}{V}$O_2peak_, significant predictors of ME_net_ were also epinephrine (ß = 0.49, *p* = 0.01), norepinephrine (ß = 0.60, *p* < 0.001) and power output (ß = 0.71, *p* < 0.001).

## Discussion

To the best of our knowledge, this study was the first to investigate the relationship between ME and many potential underlying factors among obese (OB), overweight (OW) and normal-weight (NW) adolescent boys when cycling at different exercise intensities of an incremental cycle test. Our results confirmed that (1) excess body fat had a significant effect in decreasing ME at all studied intensity levels. Moreover, (2) exercising at similar relative intensities brought about higher metabolic and physiological responses for the OB group that also presented the lowest ME values in comparison to the NW and OW groups. Finally, results showed that (3) body weight, %LO and energy consumption were significant predictors of ME_net_ at the moderate intensity (i.e., 50%HRmax), while this was no longer apparent at higher intensities where catecholamine levels and power output seemed to be better predictors of efficiency. The later finding was made possible by the choice of the studied population that presented different epinephrine and norepinephrine responses to an incremental exercise, a characteristic that was not addressed in previous studies examining factors of ME.

At rest and during all tested cycling intensity levels, absolute oxygen uptake (in L⋅min^-1^) was significantly higher for the OW and OB adolescents as compared to the NW adolescents (Nikolaidis et al., 2018), while no differences in HR values were detected between groups. This result might suggest a higher muscle oxygen extraction capacity per heart beat and/or a larger stroke volume for our obese adolescents (Salvadori et al., 1999; Lafortuna et al., 2006). The latter goes in line with previous reports on obese adult women (Lafortuna et al., 2006) and young obese adults (Salvadori et al., 1999), and could be interpreted in relation to the excess body mass and FFM. Furthermore, it has been suggested that increases in $\overset{˙}{V}$O_2_ and E during cycling in obese individuals can result from the extra work required to move the lower limbs (Anton-Kuchly et al., 1984) and the higher postural activity (Dempsey et al., 1966).

As hypothesized, obesity seems to affect metabolic and physiological responses to exercise in adolescents. Specifically, at 50%HRmax, our results showed a lower ME for the OB group in comparison to the OW and NW groups. As previously established by Butte et al. (2007), ME is negatively affected by energy expenditure rates at this intensity level in overweight children. These authors found that the higher energy expenditure in overweight children and adolescents were attributed in a large part to differences in body size and composition. Indeed, excess body weight, as presented in our OB adolescents, constitutes a major contributor for energy expenditure increases as more energy is consumed at a given work output (e.g., moderate aerobic level). Additionally, it was found that lipid oxidation rate (%LO) was a significant predictor of ME at 50%HRmax, in a way that those having a high %LO during the moderate aerobic stage were also more efficient. In fact, our OB and OW groups presented significantly higher respiratory exchange ratio values, which is a potential indicator of an impaired fat oxidation capability during exercise (van Baak, 1999). Accordingly, a recent study of Laaksonen et al. (2018) conducted in groups of subjects with different ME levels during cycling, showed a higher use of fatty acids for more efficient individuals during prolonged exercise at moderate exercise intensity. For these authors, the shift in relative contributions of fats and carbohydrates may explain the changes in ME. As assumed by Laaksonen et al. (2018), ME depends on the effectiveness of lipid oxidative capacity at moderate aerobic intensities. Despite that other complementary analyses are needed to confirm our assumption, the present study was the first to establish the link between %LO and ME at moderate aerobic intensity among obese adolescents.

At higher intensity levels (i.e., 75%HRmax and at $\overset{˙}{V}$O_2peak_), subjects’ efficiency increased as compared to its value at the moderate aerobic level (Figure 1B). This could be explained by the increases of both workload and the amount of energy consumed (Jabbour et al., 2013). At these two intensity levels (i.e., 75%HRmax and VO_2peak_), ME was negatively affected by body fat and weight status with the lowest values found for OB as compared to OW and NW adolescents. The lower ME observed for OB and OW groups may be a consequence of lower muscle performance. Accordingly, the power output developed at this stage was higher for NW as compared to OB and OW groups leading to increases in the magnitude of the numerator in the ME model, thus in the ME value. Furthermore, results revealed significantly lower epinephrine and norepinephrine responses to exercise for the OB and OW groups at all studied intensity levels. The latter supports previous findings on adolescents (Eliakim et al., 2006) showing a substantially attenuated catecholamine responses to bout of cycling exercise above the anaerobic threshold. Interestingly, the power output developed during 75%HRmax and $\overset{˙}{V}$O_2peak_ stages were significantly associated with epinephrine and norepinephrine concentrations, which was not the case at lower intensity. Indeed, as intensity increases, the reliance on fiber type-II to meet the imposed performance demand becomes greater (Sale, 1987), therefore the catecholamine responses are further stimulated by changes in acid-base balance and reduced oxygen availability to the working muscle (Schneider et al., 1992). The latter influences exercise performances by regulating muscular glycogenolysis (Richter et al., 1981).

Taken together, our results may offer a new insight in terms of ME’s assessment, especially when exploring a high intensity exercise. Actually, the inclusion of the anaerobic energy production in ME’s calculation is still unavailable and therefore not represented. Indeed, anaerobic contribution is increasingly involved in energy supply at intensities above the lactate threshold 2 (intensity corresponding to 75%HRmax in our study). This might have limited our results, especially when comparing the three groups for which the anaerobic energy contribution at 75%HRmax seemed to be the highest for the OB group (estimation simply based on lactate concentrations) potentially leading to an underestimation of ME. Moreover, ME was determined from incremental 2-min stages, which did not take into account potential differences in gas exchange kinetics between the three groups. Therefore, further studies are needed to include the anaerobic component in the determination of ME that would be examined at different steady state intensities to determine the extent of the phenomenon. Moreover, the use of net ME in this study, as opposed to gross ME, allowed us to control for group differences in baseline (i.e., resting) energy expenditure, thus increasing the reliability of ME values. However, our method of ME calculation, similar to that considering the baseline as the energy cost of unloaded cycling (i.e., work efficiency), does not take into account variations in energy expenditure required to maintain homeostasis (Moseley and Jeukendrup, 2001). This also offers perspectives into calculation methods of cycling ME in relation to differences arising from group characteristics.

## Conclusion

In conclusion, the present study highlights an important issue regarding predictors of ME in adolescent boys with different body fat percentages. It appears that underlying factors of ME may diverge according to the intensity of exercise. Our assumption of different underlying factors for ME is supported, and goes beyond the simple relation to the mass of body segments and the energy cost involved in movements (Lafortuna et al., 2006, 2009; Butte et al., 2007). Indeed, at moderate aerobic intensity, the energy consumption and lipid oxidative rates may be important factors contributing to lowering ME among obese and overweight individuals. At the contrary, at higher intensities, ME may be better explained by factors such as muscle power and catecholamine responses that are attenuated in obesity. Based on this relationship, further investigations are needed to provide a more complete profile regarding energy/metabolic forms (aerobic, anaerobic) to ensure that they are well represented in the ME model. From a practical standpoint and given the importance of ME as an indicator of exercise tolerance, it seems important to include both moderate- and high-intensity exercises to programs targeting obese adolescents, where different benefits would be expected.

## Author Contributions

GJ conceived and designed the study, collected the data, and drafted the manuscript. GJ and LM performed the data analysis and interpreted the data, and revised, read and approved the submitted version.

## Conflict of Interest Statement

The authors declare that the research was conducted in the absence of any commercial or financial relationships that could be construed as a potential conflict of interest.
